# Hyper-rapid progression in Salmonella-associated mycotic aortic aneurysms: a narrative review

**DOI:** 10.2478/raon-2026-0016

**Published:** 2026-03-24

**Authors:** Jernej Lucev, Vojko Flis, Ales Slanic, Jerneja Cujes, Silva Breznik

**Affiliations:** Department of Radiology, University Medical Centre Maribor, Maribor Slovenia; Department of Vascular and Endovascular Surgery, University Medical Centre Maribor, Maribor Slovenia; University department of Cardiology and Angiology, University Medical Centre Maribor, Maribor Slovenia

**Keywords:** mycotic aneurysm, Salmonella, visceral aorta, hyper-rapid progression, endovascular aortic repair, optimized radial sealing strategy

## Abstract

**Background:**

Mycotic aortic aneurysms (MAAs), or infective native aortic aneurysms, are rare, life-threatening infections with a high risk of rupture. Non-typhoidal *Salmonella* (NTS) species show a tropism for the diseased aorta in elderly, atherosclerotic patients, causing explosive growth poorly captured by conventional surveillance. Management is complex when the visceral/paravisceral aorta is involved, making open surgical repair (OSR) risky and requiring tailored endovascular aortic repair (EVAR). This review synthesises evidence on *Salmonella*-associated MAAs and introduces hyper-rapid progression (HRP) as an early imaging biomarker.

**Conclusions:**

Salmonella MAAs represent a high-velocity phenotype. We define HRP as progression from aortitis to saccular pseudoaneurysm (≥ 5 mm) within 7 days or rapid enlargement (≥ 5 mm or > 50%) within 72 hours despite antibiotics. HRP serves as a “red flag” for urgent mechanical stabilisation. While OSR is the gold standard, in anatomically complex or high-risk patients, complex EVAR with parallel grafts and an optimized radial sealing strategy (ORSS) offers a life-saving alternative. Prospective validation of HRP and its integration into imaging algorithms are needed to improve survival in this devastating condition.

## Introduction

Mycotic aortic aneurysms (MAAs) are an uncommon but critical subset of aortic pathology, accounting for less than 1–2% of all aortic aneurysms yet associated with markedly higher morbidity and mortality than degenerative disease.^1-5^ Historically, the term “mycotic” described the mushroom-like appearance of infected aneurysms rather than fungal aetiology. In contemporary usage, “mycotic aortic aneurysm” is used interchangeably with “infective native aortic aneurysm” and encompasses infected aortitis with either true aneurysmal dilatation or pseudoaneurysm (contained rupture)^2^; today, the vast majority of lesions are bacterial. Infection may reach the aortic wall by hematogenous seeding, contiguous spread from adjacent foci (e.g. spondylodiscitis, psoas abscess) or direct colonization of pre-existing atherosclerotic ulcers.^2,6^

Non-typhoidal Salmonella (NTS) ranks among the most frequent causative pathogens in Western countries and predominates in some Asian cohorts.^3,5,7-12^ The combination of systemic sepsis, preferential adhesion to damaged endothelium and enzymatic wall destruction predisposes to rapidly progressive, rupture-prone aneurysms, often at relatively small diameters.^8-12^ Reported mortality remains 20–40% even with treatment and approaches 80–100% without repair.^3-7^

Despite multiple observational series and systematic reviews, major uncertainties persist regarding early growth kinetics, optimal timing of intervention and the choice between open surgical repair (OSR) and endovascular aortic repair (EVAR), particularly in visceral/paravisceral disease.^1,3,4,12-20^ Existing size-based paradigms and definitions of “rapid expansion” - for example ≥ 5 mm growth in 6 months in degenerative AAA - are inappropriate for MAAs evolving over days.^21-23^ There is a pressing need for an operational concept that captures clinically meaningful short-interval progression and can be embedded into real-world workflows.

The available literature was reviewed with particular emphasis on imaging findings, growth kinetics, and management strategies in visceral and paravisceral disease.

This review synthesizes current evidence on Salmonella-associated MAAs with three specific aims:

− To summarize epidemiology, risk factors, microbiology and pathogenesis of Salmonella MAAs.
− To propose hyper-rapid progression (HRP) as an early imaging biomarker of instability, with explicit temporal and morphologic criteria.
− To discuss risk-stratified management in the context of HRP, including the advantages and limitations of OSR *vs*. EVAR in challenging visceral anatomy, illustrated with an archetypal clinical example.

## Epidemiology and risk factors

### Incidence and geographic variation

MAAs are rare, with an estimated incidence of 0.7–2.6% among all aortic aneurysms.^1,3-5^ The true burden is probably under-recognized because of pre-hospital rupture, misclassification as degenerative aneurysm and incomplete reporting.^3,6,7^

Epidemiology varies geographically. Western cohorts typically comprise elderly patients with advanced atherosclerosis and multiple comorbidities; Staphylococcus aureus and Salmonella spp. are dominant pathogens.^1,3,5,8^ In Southeast Asia, MAAs occur at younger ages and Salmonella may account for up to 50% of cases in some series, reflecting higher background rates of invasive NTS disease.^3,5,8-12^

### Predisposing patient factors

Common risk factors mirror those for invasive Salmonella infection and atherosclerotic disease:

− *Atherosclerosis*. Disrupted, lipid-laden plaques provide a favourable niche for bacterial adhesion and invasion. Salmonella MAAs are almost always superimposed on pre-existing atherosclerotic aorta.^9-11^
− *Advanced age and frailty*. Immunosenescence and multimorbidity increase susceptibility to bacteraemia and reduce physiological reserve.^8-11^
− *Immunosuppression*. Diabetes mellitus, chronic kidney disease, malignancy, HIV infection, and corticosteroid therapy are repeatedly implicated.^5,8-12^
− *Systemic NTS bacteraemia*. NTS bacteraemia in older adults with vascular disease should be considered a “red flag” for occult aortic involvement, even when initial imaging is negative.^8,11^

### Anatomical distribution

MAAs most frequently involve the infrarenal abdominal aorta (40–70%), followed by the thoracic aorta (15–25%) and iliac arteries (10–20%).^1,3-5^ Although less common, visceral and paravisceral aortic involvement (coeliac, superior mesenteric and renal segment) is particularly morbid because repair must preserve multiple high-flow branches while achieving durable control.^9,17-20^ This anatomical constraint is central to the choice between OSR and EVAR and strongly influences the risk-benefit profile of each therapy.

## Microbiology and pathogenesis of Salmonella mycotic aortic aneurysms (MAAs)

### Non-typhoidal Salmonella and vascular infection

NTS species are Gram-negative bacilli from the Enterobacteriaceae family, classically causing selflimited gastroenteritis but capable of invasive bacteraemia in vulnerable hosts. Salmonella enteritidis and Salmonella typhimurium are the most common serotypes isolated in vascular infections.^9-11^

Key virulence traits relevant to vascular invasion include^9-11^:

− *Adhesion to damaged endothelium and plaques*. Fimbriae and outer membrane proteins mediate binding to activated endothelium and the extracellular matrix of atherosclerotic plaques.^9-11^
− *Invasion of vascular smooth muscle cells (VSMCs)*. Type III secretion system (T3SS) effectors encoded on SPI-1 and SPI-2 pathogenicity islands facilitate intracellular entry into VSMCs and macrophages.^9-11^
− *Intracellular survival*. NTS can persist within macrophages and foam cells, evading antibiotics and host immunity while maintaining a chronic inflammatory milieu.^9-11^
− *Pro-inflammatory phenotype*. Infection induces high levels of TNF-α, IL-1β and IL-6, amplifying local tissue damage.^9-11^

### Mechanisms of aortic wall destruction

Development of a Salmonella MAA proceeds through several overlapping stages:

− *Seeding*. Transient or persistent bacteraemia seeds bacteria onto disrupted plaques or vasa vasorum.^2,6,8-11^
− *Local invasion*. Bacteria penetrate the media, infecting VSMCs and resident macrophages, triggering apoptosis and extracellular matrix degradation.^2,6,21-25^
− *Matrix degradation*. Upregulation of matrix metalloproteinases (MMPs), particularly MMP-9, leads to breakdown of elastin and collagen, thinning the wall and promoting pseudoaneurysm formation.^2,6,8-11^
− *Suppurative inflammation*. Dense neutrophilic and macrophage infiltration with abscess formation further weakens the wall.^2,6,26-28^
− *Outpouching and expansion*. Under systemic blood pressure, the structurally compromised wall expands outward, often in a saccular or multilobulated configuration.^2,6,29-32^

Intracellular persistence within foam cells and sustained MMP activity explain the explosive kinetics of Salmonella MAAs compared with degenerative AAAs or staphylococcal/streptococcal aneurysms, which typically enlarge over months rather than days.^6-8,33-35^

## Clinical presentation and laboratory findings

Clinical presentation is notoriously non-specific, frequently mimicking pyelonephritis, pancreatitis, spinal infection or non-specific sepsis.^1-3,6^

### Common features

− Fever and systemic inflammatory response (70–90%),
− Localized abdominal, flank or back pain,
− Elevated C-reactive protein and leukocytosis,
− Positive blood cultures in 50–85%, most commonly NTS.^3,5,8,11^

In elderly patients with NTS bacteraemia and known atherosclerosis, even subtle or transient back pain should prompt a low threshold for CT angiography (CTA). Diagnostic delay is a major driver of rupture and mortality.^3,5-8^

## Imaging modalities and diagnostic features

### CT angiography

CTA is the gold standard for diagnosis, anatomical assessment and follow-up.^1-3,6,7,23^

Aneurysms are classified as true aneurysms and false aneurysms (pseudoaneurysms). True aneurysms involve all three layers of the aortic wall (intima, media and adventitia), whereas pseudoaneurysms represent a contained rupture with blood confined by adventitia and/or surrounding tissue. In Salmonella-associated MAAs, imaging often demonstrates a pseudoaneurysmal morphology, with a focal saccular outpouching arising within an inflamed, previously normal-calibre aortic segment.

#### Typical findings

− Saccular pseudoaneurysm (contained rupture) or multilobulated aneurysmal outpouching, often eccentric.
− Rapid interval changes in size or morphology on serial imaging.
− Periaortic fat stranding, soft-tissue mass or fluid collections.
− Discontinuity of the calcified intimal rim, suggestive of penetrating infection.
− Periaortic gas in advanced or fistulising infection.
− Adjacent psoas oedema or paraspinal inflammation.

CTA is also critical for planning OSR (extent of debridement, clamp sites, bypass options) and EVAR (landing zones, branch vessel take-off, access).

### MRI

MRI is less commonly used but can help characterize soft-tissue extension, spinal involvement or differentiate subacute from chronic inflammation using T2-weighted and diffusion-weighted sequences.^23^

### 18F-FDG PET/CT and PET/MRI

18F-FDG PET/CT has emerged as an important adjunct in both diagnosis and follow-up of vascular infections.^29-31^

− *Diagnosis*. High focal FDG uptake along the aorta has high sensitivity and specificity for active aortitis or graft infection, differentiating it from non-inflamed atherosclerosis.
− *Post-intervention monitoring*. PET/CT is useful to evaluate ongoing metabolic activity around stent-grafts or prosthetic grafts, guiding duration of antibiotic therapy and detecting recurrence.
− *Limitations*. False positives may occur in vasculitis or intense atherosclerosis; interpretation must integrate clinical context.^29-31^

PET/MRI offers improved soft-tissue contrast with metabolic information and may be particularly useful in complex thoracoabdominal disease or in younger patients to reduce radiation exposure.^31^

### Natural history and growth dynamics of Salmonella mycotic aortic aneurysms (MAAs)

The natural history of MAAs is far more aggressive than that of degenerative AAAs. Multiple series report median times from symptom onset to rupture of 7–14 days, with rupture occurring at diameters less than 2 cm in some Salmonella cases.^6-8,33-35^

#### Key observations from historical and contemporary cohorts^3-8,33-35^:

− Rapid enlargement over days rather than months.
− High early rupture rate during initial sepsis.
− Significant mortality even when repaired, particularly in thoracic or visceral locations.
− Markedly worse outcomes when diagnosis is delayed or when initial imaging underestimates disease extent.

These observations challenge traditional AAA surveillance paradigms. Definitions of “rapid expansion” such as ≥ 5 mm in 6 months or ≥ 10 mm in 1 year are meaningless when Salmonella MAAs can double in size within a week.^23,33-35^ Instead, short-interval growth over 48–72 hours becomes critical for risk stratification.

## Concept and clinical rationale for hyper-rapid progression (HRP)

### Proposed definition

To standardize the description of aggressive early behavior in Salmonella MAAs, we propose hyperrapid progression (HRP) as a clinically actionable imaging biomarker defined by either of the following despite appropriate intravenous antibiotic therapy.

− *Pseudoaneurysm formation*. Progression from isolated aortitis/periaortitis (no discrete outpouching) to a measurable saccular pseudoaneurysm (≥ 5 mm bulge) within 7 days; or
− *Explosive enlargement*. An absolute increase in maximal pseudoaneurysm (sac) diameter ≥ 5 mm or a relative increase > 50% within 72 hours. These thresholds are deliberately stringent to avoid over-calling trivial measurement variability.

They were chosen based on:

− Documented cases of Salmonella MAAs forming de novo or doubling in size within 3–7 days.^33-35^
− The use of ≥ 5 mm change as a clinically significant threshold in AAA surveillance and post-EVAR sac monitoring.^23^
− Practical alignment with real-world septic workflows, where repeat CTA at 48–72 hours is often performed in unstable patients.

HRP is agnostic to absolute diameter and instead focuses on kinetics. A small but explosively enlarging 13-mm saccular lesion in the paravisceral aorta may be more dangerous than a stable 5-cm degenerative AAA.

## Clinical role of hyper-rapid progression (HRP)

− HRP is not a rigid diagnostic label but a decision-support tool that.
− Identifies patients at very high short-term risk of rupture,
− Justifies escalation from “watchful waiting” to urgent mechanical stabilization,
− Encourages routine short-interval CTA (48–72 h) in Salmonella aortitis,
− Provides a common language for multidisciplinary team (MDT) discussions and guidelines.
− In practice, HRP should be considered a strong indicator to prioritize intervention once anatomy, physiology and institutional resources are clarified - even when the lesion is small by conventional standards.

### Integrating hyper-rapid progression (HRP) into treatment pathways

HRP can be embedded into a simple, pragmatic pathway.

1. *Initial assessment*. Suspicion of MAA in a patient with NTS bacteraemia, back/abdominal pain or unexplained sepsis → urgent CTA.
2. *Baseline classification*. Aortitis/periaortitis without aneurysm *vs*. small MAA *vs*. established aneurysm.
3. Start IV antibiotics and MDT discussion.
4. *Repeat CTA at 48–72 hours* (earlier if clinical deterioration).
5. HRP assessment
  - *No signi*ficant change → continue antibiotics, plan elective OSR or EVAR according to anatomy and fitness.
  - HRP pres*ent* → treat as imminent rupture; proceed to urgent mechanical stabilisation (OSR or complex EVAR).

In this framework, HRP acts as an accelerant in the decision algorithm, especially for paravisceral aortitis where delay waiting for custom fenestrated devices may be fatal.

## Illustrative clinical scenario

A 61-year-old man with hypertension and diabetes presented with fever, abdominal and back pain, and a marked systemic inflammatory response (CRP 116 mg/L, procalcitonin *7.5* ng/mL). Blood cultures grew Salmonella enteritidis. Initial CTA showed extensive periaortic inflammation around the visceral aorta but no discrete pseudoaneurysm.

**FIGURE 1.**
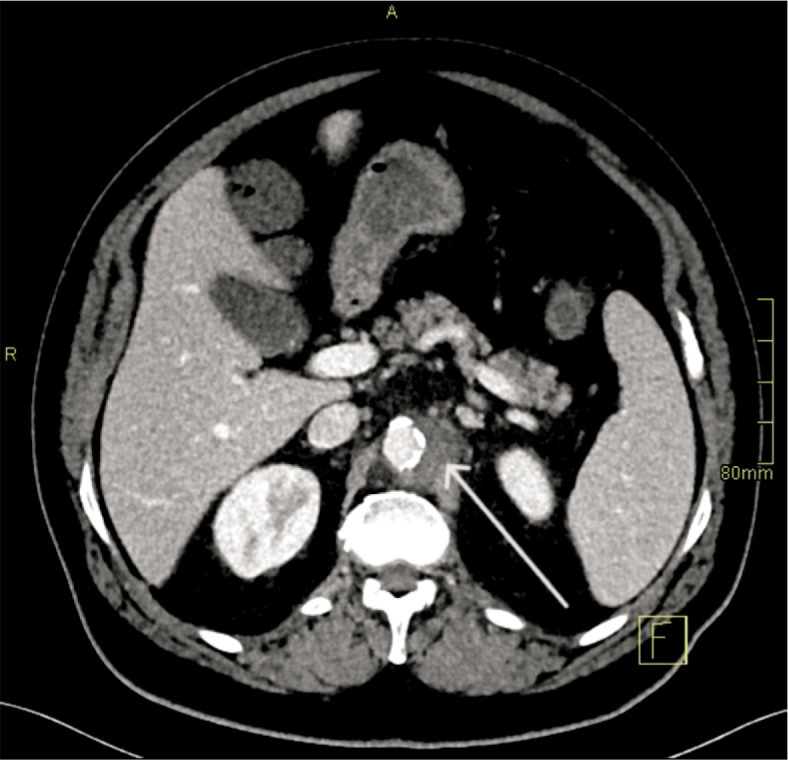
Aortitis (Day 1). Axial CT angiography (CTA) demonstrates circumferential wall thickening and periaortic fat stranding in the paravisceral aorta, without a focal outpouching (arrow).

**FIGURE 2.**
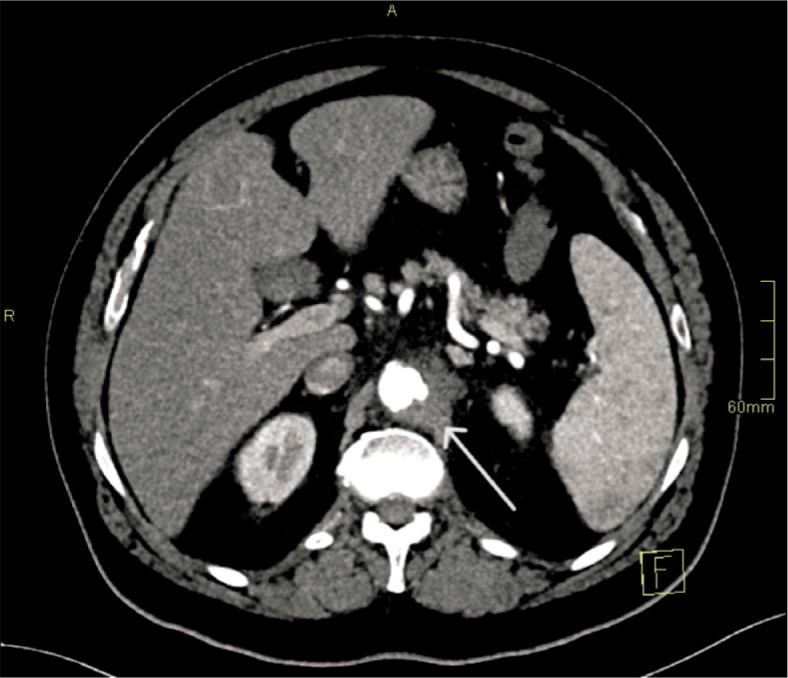
Pseudoaneurysm formation (Day 4). Axial CT angiography (CTA) demonstrates a new focal saccular pseudoaneurysm (8 × 7 mm) arising within the inflamed paravisceral segment (arrow).

**FIGURE 3.**
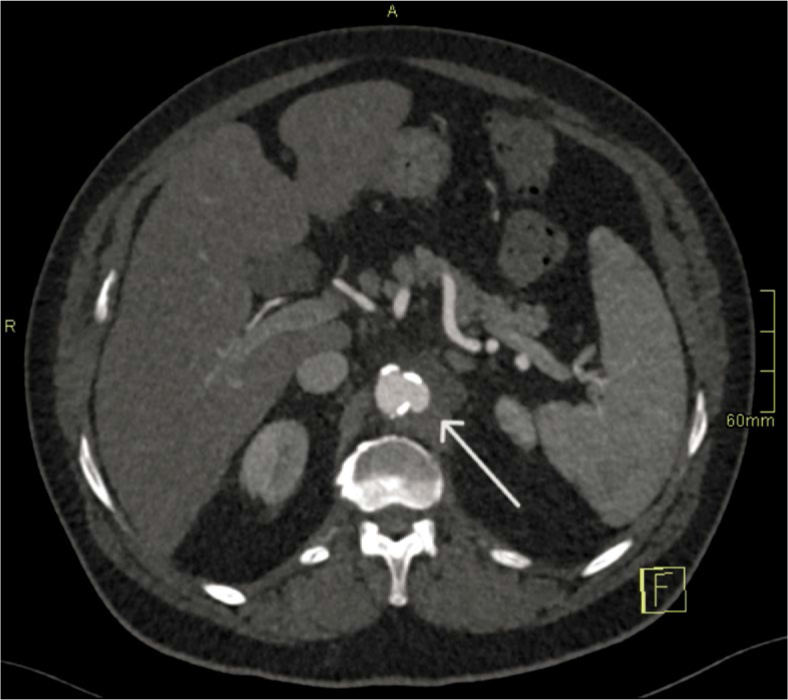
Hyper-rapid progression (Day 6–7). Axial CT angiography (CTA) demonstrates rapid expansion of the pseudoaneurysm to 13 × 7 mm over 72 hours (arrow).

− Day 1 (Aortitis). CTA demonstrated circumferential wall thickening and fat stranding from the coeliac to the renal segment, without focal outpouching. Empirical broad-spectrum IV antibiotics (ceftriaxone and flucloxacillin) were initiated and later tailored to susceptibilities (Figure 1).
− Day 4 (Pseudoaneurysm formation). Due to persistent sepsis and back pain, repeat CTA at 72 hours demonstrated a new 8 × 7 mm focal saccular pseudoaneurysm within the inflamed segment, fulfilling the first HRP criterion (*de novo* pseudoaneurysm formation within 7 days) (Figure 2).
− Day 6–7 (Explosive enlargement). A third CTA 72 hours later showed enlargement to 13 × 7 mm, representing > 60% relative increase in sac size over 3 days. This met the second HRP criterion and was interpreted as an imminent risk of rupture (Figure 3).

The pseudoaneurysm involved the paravisceral aorta with both coeliac and superior mesenteric artery (SMA) origins at the level of maximal disease and close proximity to both renal arteries. Given ongoing sepsis, paravisceral involvement and the need for prolonged supraceliac clamping and complex visceral revascularisation, the MDT judged OSR to carry prohibitive perioperative risk (high ASA and SOFA scores, poor cardiopulmonary reserve).^15,17^

Because HRP had been demonstrated, deferring intervention to await a custom fenestrated or branched EVAR device (F/B-EVAR) was deemed unsafe. An urgent off-the-shelf complex EVAR was performed using a thoracic stent-graft with parallel chimney grafts to the coeliac trunk and SMA and a periscope graft to the dominant renal artery, applying an optimized radial sealing strategy (ORSS). One accessory renal artery was sacrificed to simplify the procedure and to avoid renal artery penetration during the procedure.

**FIGURE 4.**
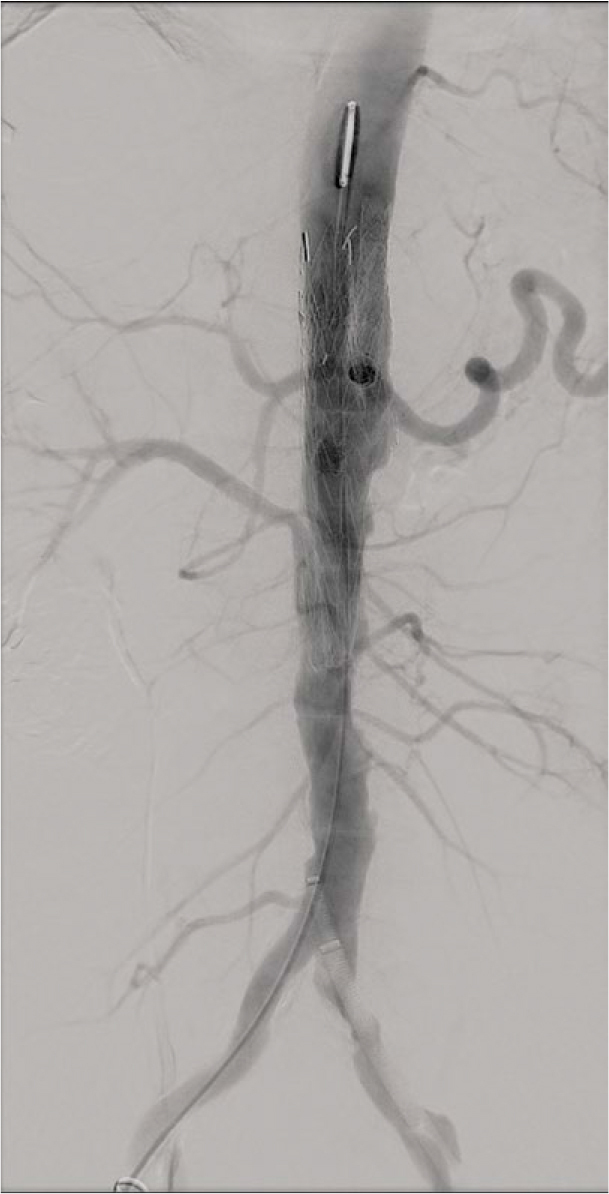
Completion angiography after complex endovascular aortic repair (EVAR). Final angiogram confirms successful exclusion of the pseudoaneurysm with preserved flow through the visceral branch stents.

**Figure 5.**
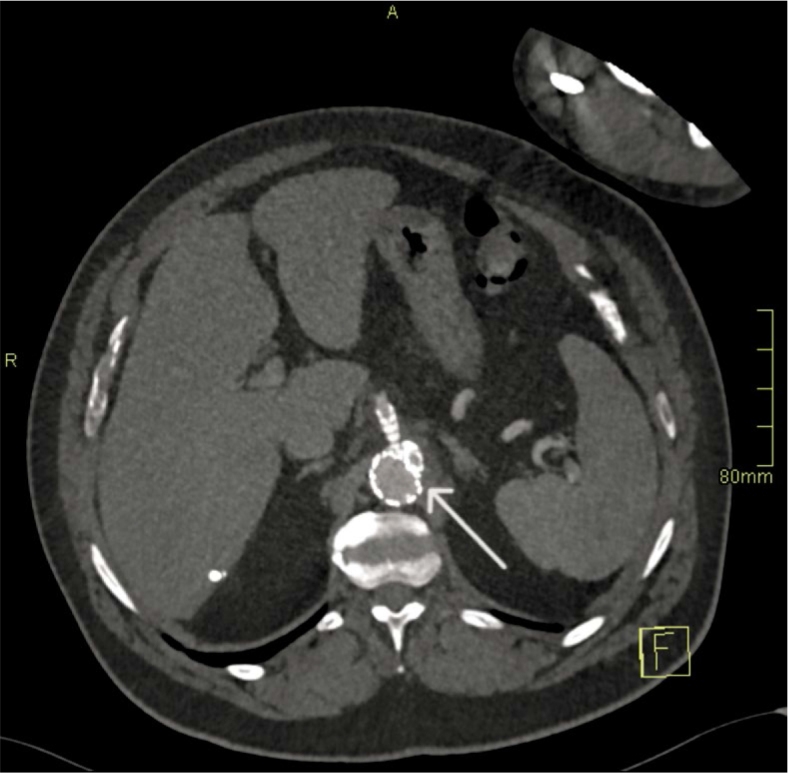
Follow-up after complex endovascular aortic repair (EVAR). Axial CT angiography (CTA) demonstrates a stable excluded sac without endoleak.

The procedure achieved immediate exclusion of the pseudoaneurysm with preserved visceral perfusion (Figure 4). Under 6 weeks of targeted IV therapy and planned lifelong suppressive oral antibiotics (agent selected by the infectious-disease team based on susceptibility) as a bridge to definitive surgery when feasible, the patient recovered without recurrent sepsis. Follow-up CTA demonstrated sac stability and no endoleak (Figure 5).

This scenario illustrates how the HRP concept can convert an ambiguous “small lesion” into a clear mandate for urgent complex EVAR when OSR is anatomically and physiologically hazardous.

## Management strategies in Salmonella MAAs

Optimal treatment requires three interdependent pillars^2,3^:

− antimicrobial therapy,
− mechanical source control (OSR or EVAR),
− structured surveillance.

### Antimicrobial therapy

Prompt initiation of broad-spectrum IV antibiotics is mandatory once MAA is suspected, tailored to culture and sensitivity results when available.^2,3,24^

For Salmonella infection, third-generation cephalosporins (e.g. ceftriaxone) and fluoroquinolones (e.g. ciprofloxacin) are preferred first-line agents due to excellent vascular penetration; carbapenems are reserved for multidrug-resistant strains.^2,8-11^ Most high-quality series recommend 4–6 weeks of IV therapy, followed by several months or lifelong oral suppression when prosthetic material remains in situ, especially after EVAR.^2-4,24-28^

In patients meeting HRP criteria, the biology implies failure of initial antimicrobial containment. Intensified therapy (e.g. combination regimens), formal infectious-disease consultation and accelerated mechanical source control should be considered.^2-4,24-28^

### Open surgical repair (OSR)

#### Advantages and indications

Comprising radical debridement of infected tissue with in situ reconstruction using cryopreserved homografts, biological grafts or antibiotic-soaked prostheses, or extra-anatomic bypass, OSR has long been considered the definitive therapy, offering^1-4,9,15,24-27^

− Direct removal of infected and necrotic tissue,
− Theoretical superior long-term microbiological cure,
− Independence from long-term stent-graft integrity.

Where infection is localized, anatomy is favoruable (e.g. infrarenal disease with adequate landing zones for clamps), and the patient is physiologically robust, OSR offers durable results with acceptable late reinfection rates.^9,15,24-27^

#### Limitations

However, OSR has important disadvantages:

− High perioperative mortality (20–40%) in contemporary series, especially in septic, elderly and comorbid patients.^3-5,9,15,24-27^
− Need for supraceliac or even supradiaphragmatic clamping in thoracic/visceral disease, with prolonged visceral ischaemia and spinal cord risk.^3-5,9,15,24-27^
− Technical complexity in paravisceral anatomy, often requiring multiple bypasses or reimplantations of coeliac, SMA and renal arteries ^3-5,9,15,24-27^
− “Hostile abdomen” after prior surgery, radiotherapy or abdominal sepsis.^3-5,9,15,24-27^

Visceral and paravisceral Salmonella MAAs exemplify the anatomical scenarios where OSR risk may outweigh benefits, particularly when HRP is present and emergent intervention is required.^3-5,9,15,24-27^

### Endovascular aortic repair (EVAR)

#### Standard EVAR

EVAR offers rapid exclusion of the aneurysm without laparotomy or aortic cross-clamping, reducing physiological stress and early mortality in suitable anatomy.^1,3,4,12-14,16,17,22,26^ Systematic reviews and multicentre series consistently show:

− Lower early mortality compared with OSR (approximately 6–10% *vs*. 15–30%),^1,3,4,12-14,26^
− Similar mid-term survival, but higher late reintervention and infection-related complication rates in EVAR cohorts.^12-14,16,17,26^
− These data support EVAR as a valuable acutephase strategy, particularly for unstable or high-risk patients.

#### Disadvantages

The main drawbacks of EVAR in infection are:

− Retention of infected aortic wall and peri-aortic tissue, with potential for persistent or recurrent infection,
− Risk of stent-graft infection, which is difficult to eradicate and often requires later explantation,
− Need for meticulous and lifelong imaging and antibiotic follow-up.^2-4,24-28^

### Complex endovascular aortic repair (EVAR) in visceral/paravisceral anatomy

For suprarenal, visceral or paravisceral MAAs, standard EVAR is usually not feasible because sealing zones would cover critical branches. Advanced options include^18-21,26,27^:

− *Fenestrated/branched EVAR (F/B-EVAR)*. Custommade devices with fenestrations or side branches for coeliac, SMA and renal arteries provide anatomical perfection but require weeks for manufacture, an unacceptable delay in HRP-positive Salmonella MAAs.
− *Parallel graft techniques (chimney/periscope EVAR)*. Off-the-shelf thoracic or abdominal stent-grafts combined with parallel covered stents to visceral branches. These can be deployed urgently and are pivotal in the HRP setting.^19,20^

### Optimized radial sealing strategy (ORSS)

Parallel-graft EVAR introduces the risk of “gutter leaks” between the main stent-graft and chimney/periscope grafts. In the context of an infected sac, even small gutters may perpetuate pressurisation and provide a niche for bacteria.^19-21^

An optimized radial sealing strategy (ORSS) aims to minimize this risk by^19-21^:

− *Aggressive oversizing* of the main aortic stentgraft by 20–30% relative to the healthy aortic diameter to maximise radial force and apposition.
− *Generous overlap* (more than 2–3 cm) between chimney/periscope grafts and the main stentgraft to enhance stability and sealing.
− *Coordinated kissing balloon inflation* to mould all components simultaneously and reduce gutter size.
− *Adjunct sac management*, including coil or plug embolization in selected cases with incomplete sac regression or residual perfusion.

ORSS does not eliminate infection risk but offers a technically reproducible approach to maximizing mechanical sealing in carefully selected patients when complex EVAR is the only realistic option in HRP-positive settings.

### Comparative advantages and limitations of Open surgical repair (OSR) vs. endovascular aortic repair (EVAR)

Taken together, these data support a **risk-adapted strategy**. OSR for fit patients with favourable anatomy and no HRP, and EVAR (including complex parallel-graft approaches with ORSS) for unstable, HRP-positive or anatomically hostile cases where OSR is unsafe (Table 1, Table 2).^1,3,4,12-14,16,17,26^

**TABLE 1.** Analysis of open surgical repair

Category	Description
**Advantages of OSR**	**Radical debridement & cure:** allows for radical debridement and offers the potential for a microbiological cure. **Avoidance of foreign material:** avoids leaving long-term foreign material in an infected field, especially when biological grafts or homografts are used. **Lower reinfection risk:** demonstrated lower late reinfection risk in several case series.
**Limitations of OSR**	**High perioperative mortality:** associated with high mortality rates during and immediately after surgery, particularly in patients who are septic, frail, or physiologically unstable. **Technical complexity:** technically demanding procedure, especially involving thoracic and paravisceral disease, often resulting in prolonged ischaemia. **Feasibility issues:** often not feasible for High-Risk Patients (HRP) where immediate treatment is critical; delays or prolonged operations in these cases can be fatal.

The following table outlines the key advantages and limitations associated with open surgical repair (OSR), particularly in the context of complex vascular conditions and infection management.

**TABLE 2.** Analysis of endovascular aortic repair (EVAR)/Complex EVAR

Category	Description
**Advantages of EVAR**	**Minimally invasive:** rapid stabilization without laparotomy or aortic clamping. **Lower Early Mortality:** consistently shown in multiple series and systematic reviews. **High-Risk suitability:** particularly attractive for High-Risk Patients (HRP), those with haemodynamic instability, hostile abdomen, or high surgical risk. **Visceral perfusion:** parallel-graft techniques allow for urgent preservation of visceral perfusion in anatomically complex lesions.
**Limitations of EVAR**	**Long-term complications:** higher rates of late reinfection, sac enlargement, or endoleak compared with OSR. **Technical & device issues:** complex parallel-graft constructs carry risks of gutter endoleaks and device fatigue. **Maintenance:** requires lifelong reliance on imaging follow-up and antibiotic suppression.

The following table details the advantages and limitations of Endovascular endovascular aortic repair (EVAR) and its complex variations.

## Future directions and unanswered questions

Despite increasing experience, several critical gaps remain:

− *Validation of HRP*. Prospective multicentre studies are needed to confirm the prognostic value of the proposed temporal and morphologic thresholds and to refine absolute *vs*. relative growth criteria.^33-35^
− *Biomarkers of “molecular cure”*. Novel biomarkers, including PCR-based pathogen load, cell-free microbial DNA and inflammation-linked signatures, may help determine when infection has truly resolved and guide duration of antibiotic therapy.^32^
− *Role of PET/CT and PET/MRI*. Standardized imaging protocols and interpretation criteria are required to integrate metabolic imaging into routine follow-up algorithms after EVAR or OSR.29-31
− *Device innovation*. Rapid-manufacture customized F/B-EVAR platforms or off-the-shelf branched devices may offer safer alternatives to parallel-graft techniques in the HRP setting. ^29-31^
− *Guideline integration*. If validated, HRP should be incorporated into future guidelines as a red-flag metric, triggering short-interval CTA and MDT review in all Salmonella aortitis cases.

## Conclusions

Salmonella-associated mycotic aortic aneurysms are a high-lethality, high-velocity phenotype of aortic infection with rapid early progression and a high risk of rupture-especially in visceral and paravisceral locations-making conventional sizebased thresholds inadequate.^1-3^

We propose hyper-rapid progression (HRP) as an imaging biomarker of imminent instability, defined by either de novo saccular pseudoaneurysm/aneurysmal outpouching formation (≥ 5 mm) within 7 days, or explosive enlargement (≥ 5 mm or > 50%) within 72 hours despite appropriate antibiotics. OSR remains the reference standard for durable source control when feasible, but in HRP-positive, unstable or anatomically complex disease, urgent complex EVAR (including parallel-graft techniques with an Optimized Radial Sealing Strategy) may be life-saving, at the cost of higher late infection-related risks and the need for meticulous, lifelong surveillance.^1-5,36^
